# A Novel Method for Separating and Locating Multiple Partial Discharge Sources in a Substation

**DOI:** 10.3390/s17020247

**Published:** 2017-01-27

**Authors:** Pengfei Li, Wenjun Zhou, Shuai Yang, Yushun Liu, Yan Tian, Yong Wang

**Affiliations:** 1 School of Electrical Engineering, Wuhan University, No.299, Bayi Road, Wuhan 430072, China; pengfei9966@126.com (P.L.); wjzhou@whu.edu.cn (W.Z.); silencelys@163.com (Y.L.); 2 Guangzhou Power Supply Bureau Co. Ltd., No.38, Huangshidong Road, Guangzhou 510620, China; babyyan22@163.com (Y.T.); wangy@gzpsc.com (Y.W.)

**Keywords:** substation, partial discharge localization, multiple PD sources separation, multi-point measuring direction, error probability

## Abstract

To separate and locate multi-partial discharge (PD) sources in a substation, the use of spectrum differences of ultra-high frequency signals radiated from various sources as characteristic parameters has been previously reported. However, the separation success rate was poor when signal-to-noise ratio was low, and the localization result was a coordinate on two-dimensional plane. In this paper, a novel method is proposed to improve the separation rate and the localization accuracy. A directional measuring platform is built using two directional antennas. The time delay (TD) of the signals captured by the antennas is calculated, and TD sequences are obtained by rotating the platform at different angles. The sequences are separated with the TD distribution feature, and the directions of the multi-PD sources are calculated. The PD sources are located by directions using the error probability method. To verify the method, a simulated model with three PD sources was established by XFdtd. Simulation results show that the separation rate is increased from 71% to 95% compared with the previous method, and an accurate three-dimensional localization result was obtained. A field test with two PD sources was carried out, and the sources were separated and located accurately by the proposed method.

## 1. Introduction

Partial discharge (PD) detection and localization in a substation contribute to the assessment of high-voltage equipment insulation condition efficiently [1]. When PD occurs inside a piece of equipment, the ultra-high frequency (UHF) signal can radiate to the external space through any non-metallic shield regions [2]. These regions can be located by the UHF method [1,2]. In a substation, the PD may occur in multi-equipment simultaneously, and the UHF signals are always interfered by the field noise [3], which makes it difficult to locate the PD sources. Therefore, it is necessary to research the separation and localization method of multi-PD sources in the substation site.

In order to locate multi-PD sources in a substation, the sources should first be separated. Several researches on the separation have been carried out. A wireless sensor network was used to locate multi-PD sources based on received signal strength indicator [4]. However, it is difficult to obtain accurate signal strength because of the multipath effect [5] and the field noise. The spatial spectrum estimation was adopted to estimate the directions of the multi-PD sources in free space [6]. However, with the directions on two-dimensional (2-D) plane, it is difficult to locate the PD source in substation. In research by Hou et al. [7], the five frequency points of a set of UHF signals, which correspond to the maximum difference of the different PD sources, were used as the characteristic parameters to separate the multi-PD sources. The location of the PD sources was obtained based on the time difference of arrival (TDOA) method by the antenna array composed of four omnidirectional antennas. However, if the PDs occurred in the same insulated dielectric, the radiated UHF signals have a similar spectrum [8]. Furthermore, due to field noise interference and multipath effect [5,9], the difference of the five characteristic parameters may be distorted, leading to reduced separation rate [7]. In the meantime, the localization result has a low accuracy with the coordinates on the 2-D plane, due to the limitation of the sampling rate [10,11]. According to previously reported results [1,2,7,12,13,14], the localization result were all coordinates or directions on the 2-Dplane.

The locations of multi-PD sources are always different; therefore, the time delay (TD) of the two UHF signals received by any two antennas of the antenna array are different. With a different array model, the TD sequence has different distribution features. Using the TD distribution feature, the multi-PD sources can be separated. As found in [7,15,16], the probability of UHF signals radiated from multiple PD sources overlapping each other are very small. Therefore, each group of the UHF signals recorded by the acquisition system should be taken as a single PD source. Taking TD sequence as the characteristic parameter, the separation and localization of multi-PD sources can be realized.

In this paper, a novel method for separating and locating multi-PD sources is proposed. Two directional antennas, signal acquisition system and some accessories are used as the direction measuring platform. By rotating the platform at different angles at a fixed measurement point, the TD sequences are obtained. The relationship between TD and rotating angle are then analyzed, and used to separate the multi-PD sources. With the separated TD sequence, the direction angle between the measurement point and PD source can be calculated in 3-D. With measurement taken at multiple points, the PD can be located in 3-D by the multi-directions based on the error probability localization method. To verify the method, a simulated model with three PD sources is established using the software package XFdtd, the separation rate and localization are examined at a range of signal-to-noise ratios (SNR), and compared with the previously reported method. A field test in a 220 kV substation with two PD source is then presented. The effectiveness of the method is verified by the simulation and field test.

## 2. The Multi-PD Sources Separation Method Based on the TD Distribution Feature

### 2.1. The Direction Measurement Method in 3-D for One PD Source

**Figure 1:**
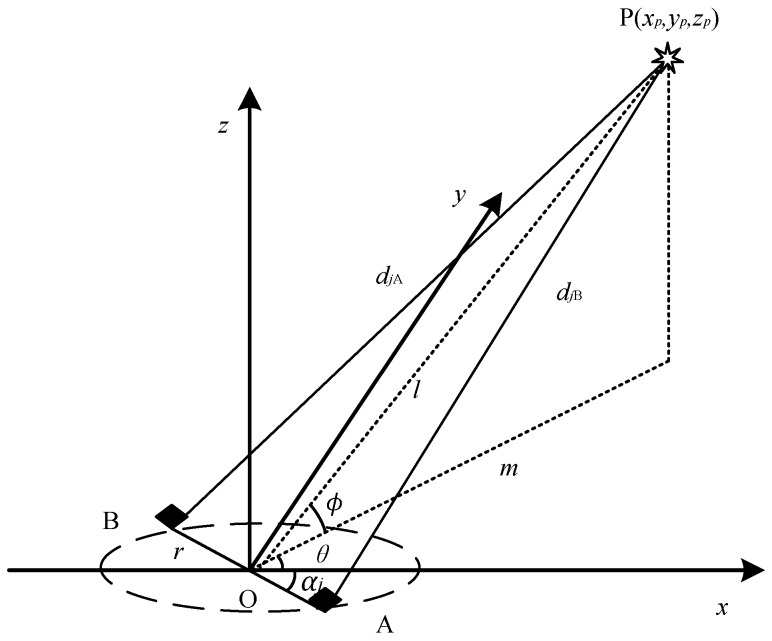
The direction measurement method for one partial discharge (PD) source based on two directional antennas.

During the PD localization experiment in a substation, the substation is considered as a limited half space, *z* ≥ 0, as shown in Figure 1. The measurement system in the proposed method is composed of two directional antennas, a rotating rod and a data acquisition system. In Figure 1, the original point *o* coincides with the measurement point O, and the two directional antennas are located at both ends of the rod AB which can be rotated around the middle point O freely on the *xoy* plane, where OA = OB = *r*, *α_j_* is the rotating angle, *θ* and *φ* are the direction angles between the PD source and the measurement point, and *l* is the distance between measuring point and the PD source. The coordinate of assumed PD source P is (*x_p_*,*y_p_*,*z_p_*). The UHF signals are received by the antennas through the paths PA and PB.

There is a TD Δ*t* between the UHF signals received by the two antennas, due to the difference in length of the path PA and PB. By rotating the platform, all equipment in the substation is covered. The TD sequence Δ*t_j_* is obtained by rotating the platform. The relationship between Δ*t_j_* and the paths is shown in Equation (1), where *d_j_*_A_ and *d_j_*_B_ are the length of PA and PB, *c* is the propagation velocity of the electromagnetic wave in air, *c* ≈ 3.0 × 10^8^ m/s. According to geometrical relationship, *d_j_*_A_ and *d_j_*_B_ are calculated by Equation (1).

To obtain the coordinate of a PD source in 3-D, at least three equations are needed, as shown in Equation (2). The subscripts of Δ*t* 1, 2, 3 represent three TDs obtained at three different rotating angles. The TD is calculated based on the UHF signals recorded by the data acquisition system. According to reference [10,11], the sampling rate of the current acquisition system is too low to locate the PD source. The solution P’(*x*’*_p_*,*y*’*_p_*,*z*’*_p_*,) of Equation (2) is not the coordinate of the PD source. However, the direction of the vector **OP’** is equal to the direction of **OP**. With the method, the direction angles of the PD source (*θ*, *φ*) can be obtained by Equation (3).

(1) $$\left\{ \begin{array}{l} d_{jA}-d_{jB}=c\cdot \Delta t_{j}\\ d_{jA}=\sqrt{{\left(x_{p}-r\cos\alpha_{j}\right)}^{2}+{\left(y_{p}+r\sin\alpha_{j}\right)}^{2}+z_{p}^{2}}\\ d_{jB}=\sqrt{{\left(x_{p}+r\cos\alpha_{j}\right)}^{2}+{\left(y_{p}-r\sin\alpha_{j}\right)}^{2}+z_{p}^{2}} \end{array}\right.$$

(2) $$\left\{ \begin{array}{l} d_{1A}-d_{1B}=c\Delta t_{1}\\ d_{2A}-d_{2B}=c\Delta t_{2}\\ d_{3A}-d_{3B}=c\Delta t_{3} \end{array}\right.$$

(3) $$\left\{ \begin{array}{l} \theta =\arctan(\frac{y_{p}^{\prime }}{x_{p}^{\prime }})\\ \varphi =\arctan(\frac{z_{p}^{\prime }}{\sqrt{{\left(x_{p}^{\prime }\right)}^{2}+{\left(y_{p}^{\prime }\right)}^{2}}}) \end{array}\right.$$

### 2.2. The Multi-PD Sources Separation Characteristic Parameter

From Section 2.1, during PD measurement by rotating the platform, the TD changes with the rotating angles, and the relationship between Δ*t_j_* and *α_j_* is shown in Equation (4), where $m=\sqrt{x_{p}^{2}+y_{p}^{2}}$, *l* = $\sqrt{x_{p}^{2}+y_{p}^{2}+z_{p}^{2}}$, $\cos\theta =\frac{x_{p}}{m}$, and $\sin\theta =\frac{y_{p}}{m}$. As *l*^2^ + *r*^2^ ≥ 2*lr*, and *l* > *m*, so, *l*^2^ + *r*^2^ > 2*mr*. It indicates that $\left|\frac{2mr}{l^{2}+r^{2}}\cos(\alpha_{j}+\theta )\right|<1$, and the Equation (4) can be simplified by the Thaler series expansion, as shown in Equation (5).

(4) $$\begin{array}{l} c\cdot \Delta t_{j}=d_{jA}-d_{jB}\\ \;=\sqrt{{\left(x_{p}-r\cos\alpha_{j}\right)}^{2}+{\left(y_{p}+r\sin\alpha_{j}\right)}^{2}+z_{p}^{2}}-\sqrt{{\left(x_{p}+r\cos\alpha_{j}\right)}^{2}+{\left(y_{p}-r\sin\alpha_{j}\right)}^{2}+z_{p}^{2}}\\ \;=\sqrt{{\left[m-r\cdot \cos(\alpha_{j}+\theta )\right]}^{2}+r^{2}\sin^{2}(\alpha_{j}+\theta )+z_{p}^{2}}-\sqrt{{\left[m+r\cdot \cos(\alpha_{j}+\theta )\right]}^{2}+r^{2}\sin^{2}(\alpha_{j}+\theta )+z_{p}^{2}}\\ \;=\sqrt{l^{2}+r^{2}}(\sqrt{1-\frac{2mr}{l^{2}+r^{2}}\cos(\alpha_{j}+\theta )}-\sqrt{1+\frac{2mr}{l^{2}+r^{2}}\cos(\alpha_{j}+\theta )}) \end{array}$$

(5) $$\begin{array}{l} \Delta t_{j}=\frac{\sqrt{l^{2}+r^{2}}}{c}(\sqrt{1-\frac{2mr}{l^{2}+r^{2}}\cos(\alpha_{j}+\theta )}-\sqrt{1+\frac{2mr}{l^{2}+r^{2}}\cos(\alpha_{j}+\theta )})\\ \;=\frac{\sqrt{l^{2}+r^{2}}}{c}\{\frac{2mr}{l^{2}+r^{2}}\cos(\alpha_{j}+\theta )+\frac{1}{8}{\left[\frac{2mr}{l^{2}+r^{2}}\cos(\alpha_{j}+\theta )\right]}^{3}+R_{n}[\frac{2mr}{l^{2}+r^{2}}\cos(\alpha_{j}+\theta )]\} \end{array}$$

In order to make the measurement easy, the height of the platform was made lower than the height of the equipment *z_p_*. During the PD measurement, it is necessary to keep a safe distance between the platform and the equipment; hence, the rotary rod should not be too long. In this paper, *r* was set to 0.75 m [11]. Taking the 220 kV substation as an example, the safe distance was 3 m, and $\left|\frac{2mr}{l^{2}+r^{2}}\right|\leq \frac{1}{2}$, when *r* = 0.75m, *m* ≥ 3 m, and *z_p_* ≥ 0 m. The Equation (5) can be simplified as given in Equation (6).

(6) $$\begin{array}{l} \Delta t_{j}\geq \frac{2mr}{c\sqrt{l^{2}+r^{2}}}\cos(\alpha_{j}+\theta )[1+\frac{1}{8}\cdot \frac{1}{4}\cos^{2}(\alpha_{j}+\theta )]+R_{n}[\frac{2mr}{l^{2}+r^{2}}\cos(\alpha_{j}+\theta )]\\ \;\approx \frac{2mr}{c\sqrt{l^{2}+r^{2}}}\cos(\alpha_{j}+\theta ) \end{array}$$

Equation (6) indicates that the maximum difference between Δ*t_j_* and $\frac{2mr}{c\sqrt{l^{2}+r^{2}}}\cos(\alpha_{j}+\theta )$ is $\frac{1}{32}\cdot \frac{2mr}{c\sqrt{l^{2}+r^{2}}}\cos^{3}(\alpha_{j}+\theta )$, when *z_p_*= 0 m, *m* = 3 m and *α_j_*+ *θ* = 0°. However, by properly designing the platform and selecting the measurement points, the *z_p_*> 0 m and *m* > 3 m. When *α_j_*+ *θ* = 0°, the antenna gain of the directional antenna is very small, the UHF signal cannot be received when setting the trigger type of the data acquisition system as width trigger. Therefore, the TD sequence distribution feature can be defined as in Equation (7), where Φ is the beam-width of the antenna. It is clear that the relationship between the TD sequence and the rotating angle satisfies the cosine function.

(7) $$\Delta t_{j}\approx \left\{ \begin{array}{l} \frac{2mr}{c\sqrt{l^{2}+r^{2}}}\cos(\alpha_{j}+\theta )\;if\;90{}^{\circ}-\frac{\Phi }{2}<(\alpha_{j}+\theta )<90{}^{\circ}+\frac{\Phi }{2}\\ \mathrm{NULL}\;else \end{array}\right.$$

Let $\gamma =\frac{2mr}{c\sqrt{l^{2}+r^{2}}}$; the coefficients *γ* and *θ* reflect the feature of the TD sequence distribution. If there are multi-PD sources in the substation, *γ* and *θ* can be used as the characteristic parameters to separate the TD sequences caused by the multi-PD sources.

### 2.3. The Direction Separation Method of Multi-PD Sources

**Figure 2:**
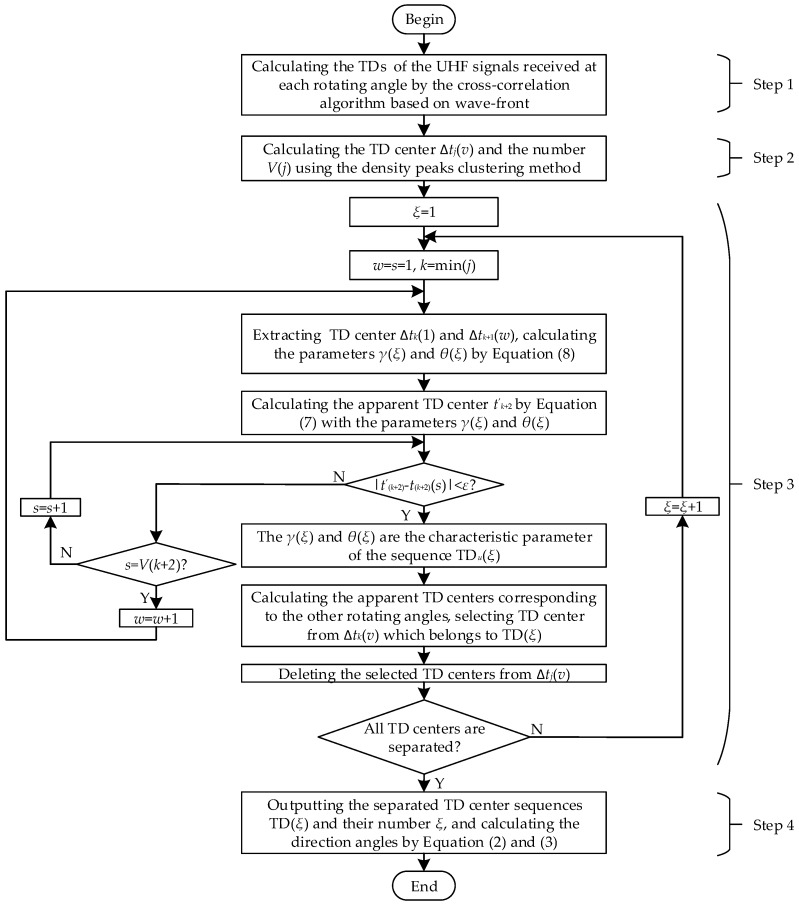
The separation method of the time delay (TD) sequences.

Assume that *V* PD sources exist in a substation. The directions separation procedure is shown as Figure 2, and the procedure is described as follows:

Step 1: Signal acquisition and TD estimation. The platform is rotated and measurements taken at several angles. At each rotating angle, *I* groups UHF signals *x_i_*_A_(*t*) and *x_i_*_B_(*t*) are recorded (*i* = 1, 2, 3, …, *I*, *I* > 100). Then, the TDs are calculated by the correlation algorithm based on wave-front of the UHF signal [11]. The TD estimation method is divided into two parts. Firstly, the wave front *x_i_*_Awf_(*t*) and *x_i_*_Bwf_(*t*) are extracted from the UHF signals. It can be considered that there is only a TD and an amplitude difference between *x_i_*_Awf_(*t*) and *x_i_*_Bwf_(*t*). Secondly, the TD is calculated by generalized cross-correlation algorithm.

Step 2: Extracting the TD center. At each rotating angle, *I* TDs are obtained, which are radiated by the *V* PDs or less. Theoretically, if the location of the PD source is fixed, the value of TD is a fixed constant when the measurement platform is rotating at a fixed angle. In reality, due to the limitation of sampling rate and low SNR, the calculated TDs may not be a fixed value. The TDs distribute near a fixed value, which is the TD center. The TD center Δ*t_j_*(*v*) and its number *V*(*j*) are obtained by the density peaks clustering method [16] (as described in Section 2.4).

Step 3: Separating the TD center sequences. The separation process is given in Figure 2. Two TD centers Δ*t_k_*(1) and Δ*t_k_*_+1_(1) are selected from the rotating angles *θ_k_* and *θ_k_*_+1_, and they are used to calculate the characteristic parameters of *γ*(*ξ*) and *θ*(*ξ*) by Equation (8), where *ξ* is the current number of TD sequence. Then the TD center of the remaining rotating angles with *γ*(*ξ*) and *θ*(*ξ*) is selected, which belongs to the TD sequence TD(*ξ*). The selected TDs are deleted and the parameters *k* and *ξ* re-assigned. The process is then repeated until all TD centers are separated. In the end, the maximum of *ξ* is the number of the TD sequence.

(8) $$\left\{ \begin{array}{l} \gamma \cos(\alpha_{1}+\theta )=\Delta t_{1}\cdot c\\ \gamma \cos(\alpha_{2}+\theta )=\Delta t_{2}\cdot c \end{array}\right.$$

Step 4: Calculating the direction between the PD source and the measurement point. With the separation results, any three TD centers are extracted from each TD center sequence and the direction calculated by Equations (2) and (3).

### 2.4. The TD Center Estimation Usingthe DensityPeaks Clustering Method

The TD values corresponding to multi-PD sources at a rotating angle have two features. The first is that the TDs of a PD source have a higher local density than their neighbors. The second is that the distance between the two TD centers is much larger than the distance between two TDs of a PD source. By calculating the local density and distance, the number and the value of the TD center can be obtained. The procedures are described as follows [17]:

Step 1: Calculating the Euclidean distance as Equation (9), where *k* ∈ 1,2,3, …, *I*, …, I, and *i ≠ k*.

(9) $$d_{ik}=\left|\Delta t_{ji}-\Delta t_{jk}\right|$$

Step 2: By Equation (10), the local density *ρ_i_* is calculated, where *d_c_* is a cutoff distance. According to [17,18], *d_c_* = 2%max(*d_ik_*). The meaning of *ρ_i_* is that the greater the number of TD samples whose distance to Δ*t_ji_* smaller than *d_c_*, the larger of the density is.

(10) $$\rho_{i}=\sum_{k\in I\backslash \{i\}}e^{-{\left(\frac{d_{ik}}{d_{c}}\right)}^{2}}$$

Step 3: The distance *δ_i_* is obtained by Equation (11); the value of *δ_i_* is taken as the minimum of the Euclidean distance between the TD sample whose local density is larger than Δ*t_ji_* and Δ*t_ji_*.

(11) $$\delta_{i}=\left\{ \begin{array}{l} \min_{k:\rho_{k}>\rho_{i}}(d_{ik})\;if\;\exists \;k\;s.t.\rho_{k}>\rho_{i}\\ \max_{k:\rho_{k}>\rho_{i}}(d_{ik})\;else \end{array}\right.$$

Step 4: Taking *ρ_i_* and *δ_i_* as the horizontal and vertical coordinates respectively, which is the decision diagram. The TD sample of which both the local density and distance are the maximum is the TD center.

During the PD measurement experiment, there may be some singular values in TDs. The reason is that the SNR is too low or the UHF signal is not detected because of the acquisition system being triggered by the field noises. By the proposed method, the distances of the singular TDs may be large, but the densities are small. With the proposed decision diagram, these singular TDs can be excluded.

## 3. The Multi-PD Sources Localization Method Based on Error Probability

**Figure 3:**
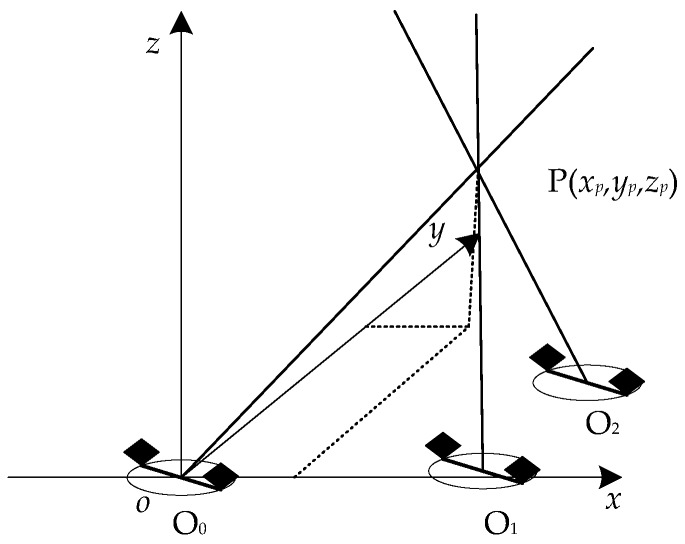
The localization principle for one PD source with multi-PD source.

In order to obtain the coordinate of the multi-PD sources in 3-D, multiple measurement points are selected in a substation, and the same number of directions can be obtained. The localization method is shown in Figure 3. Assuming that three measurement points are selected, the three directions can ideally intersect at the PD source. However, due to the resolution of sampling rate and coordinate measuring errors, these lines are skew lines. As a result, there may be no intersection in 3-D. In the field of statistics, the errors of TD and coordinates of the measurement points have normal distribution; therefore, the errors of the directions also have normal distribution. The root mean square error (RMSE) of *θ* and *φ* can be used to determine the accuracy of the localization result. The probability *p_i_*(*θ*, *φ*) of every point in 3-D is calculated by Equation (12), where *σ_θ_* and *σ_φ_* are the RMSE introduced by TD errors, coordinate measurement error and the accuracy of the measurement platform. (In this paper, the RMSEs are obtained through experimental statistics. Through a large number of tests, the RMSEs of *θ* and *φ* are obtained as *σ_θ_* = 0.79° and *σ_φ_* = 3.98°, respectively). Therefore, the PD source may be located at the point whose probability is the minimum of the joint probability density function *p*(*θ*, *φ*). The function is defined as Equation (13). With the localization method, the PD source can be located in 3-D accurately.

(12) $$p_{i}(\theta ,\phi )=1-\frac{1}{\sqrt{2\pi \sigma_{\theta }\sigma_{\phi }}}\exp\left\{-\frac{1}{2}[\frac{{\left(\theta -\theta_{Oi}\right)}^{2}}{\sigma_{\theta }^{2}}+\frac{{\left(\phi -\phi_{Oi}\right)}^{2}}{\sigma_{\phi }^{2}}]\right\}$$

(13) $$p(\theta ,\phi )=\prod_{i=1}^{I}p_{i}(\theta ,\phi )$$

If there are multi-PD sources in the substation, the directions should be separated before calculating the coordinates of the PD sources. The separation method is described as follows, assuming that there are *V* PD sources in a substation, and *U* measurement points are selected.

Step 1: All directions are used to calculate the error probability using Equations (12) and (13), and the coordinate of the minimum is obtained P′*_v_* (*x*_P*v*_, *y*_P*v*_, *z*_P*v*_).

Step 2: The apparent directions (*θ*′***_uv_***,*φ***′*_uv_***) between P′*_v_* (*x*_P*v*_, *y*_P*v*_, *z*_P*v*_) and the measurement points are calculated. If |*θ*′*_uv_* − *θ_uv_*| < 2·*σ_θ_* and |*φ*′*_uv_*−*φ_uv_*| < 2·*σ_φ_*, the direction (*θ_uv_*,*φ_uv_*) is selected. Using this judgment method, the directions corresponding to the PD source *v* are obtained.

Step 3: Repeating Step 1 and Step 2 with the unselected directions, the remaining PD sources can be located.

With the separated directions, the multi-PD sources can be located by Equations (12) and (13).

## 4. Verification of Simulation

### 4.1.The Simulation Arrangement

**Figure 4:**
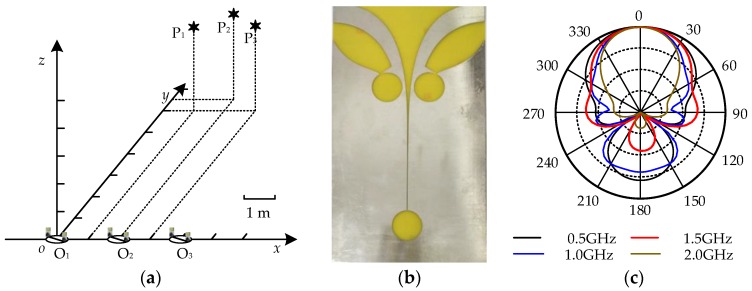
The simulation model arrangement and the Vivaldi antenna; (**a**) The space distribution of the simulated PD sources and the measurement points; (**b**) The Vivaldi antenna; (**c**) The radiation pattern of the Vivaldi antenna.

To verify the proposed method, XFdtd version 7.3 was utilized to simulate UHF signals radiated by PDs in a substation. A 3-D coordinate system was established in the simulated substation space, as shown in Figure 4a. For comparison with the previously reported method [7], the arrangement of the simulation was the same as in the literature [7]. Three simulated PD sources were set, and the coordinates were P_1_ (2, 6.5, 3), P_2_ (3, 6, 3), and P_3_ (1, 6, 3) in meters. The simulated PD sources were generated by current elements which are Gaussian impulses. Three measurement points were selected at O_1_ (0, 0, 0), O_2_ (2, 0, 0), O_3_ (4, 0, 0), and *r* = 0.75 m. The directional antenna is the Vivaldi antenna [19] as shown in Figure 4b. The band-width is 0.5–3 GHz, and the radiation pattern is shown in Figure 4c. The rotating angle is from −45° to 45°.The two groups of parameters of the three simulated PD sources are provided, as shown in Table 1. The parameters in Case 1 are the same as the previously reported method. The parameters in Case 2 are different from the previously reported method, and Case 2 is used to verify the effectiveness of the two methods with the same PD source.

### 4.2. The Simulation Result of Case 1

**Figure 5:**
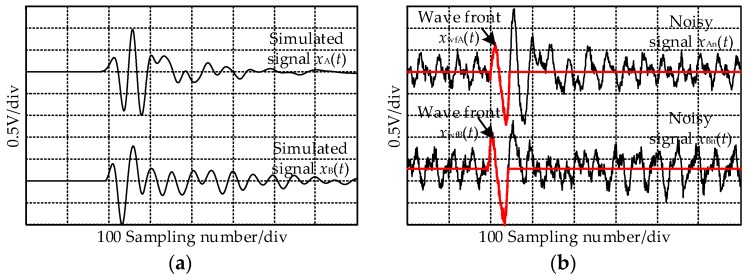

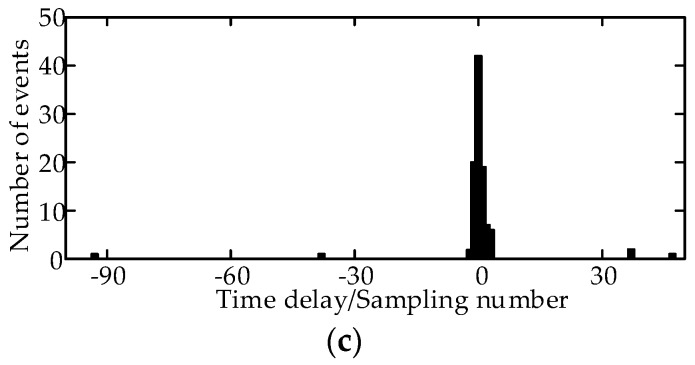
The original and noisy ultra-high frequency (UHF) signals radiated by P_2_ at O_2_ and the calculated TDs. (**a**) The received UHF signals radiated by P_2_ at O_2_; (**b**) The noisy UHF signals when the signal-to-noise ratio (SNR) = 5 dB; (**c**) The calculated TDs.

The normalized UHF signals radiated by a PD source P_2_ at the measurement O_2_ (Figure 4) when the rotating angle is 0° are listed in Figure 5a, and the sampling rate is 40 GS/s. To simulate the field noise interference, the received signals were added with Gaussian white noise and communication frequency interference of different SNRs, and the frequencies were 900 MHz and 1800 MHz. For different SNRs, 100 signals of each type were obtained. To make sure that the simulation reflects the actual situation, different white noise and random phase communication frequency interference as added to each signal. When SNR = 5 dB, the value of the white noise was 0.08 V, the values of the communication frequency interference were 0.3 V and 0.15 V. The noisy UHF signal is shown in Figure 5b. Using the TD estimation method given in appendix, the wave-fronts were obtained as in Figure 5b, and the calculated TDs are shown in Figure 5c. The majority TDs gathered near 0, and the minorities have singular values. The singular value is not the real TD, the reason for which is that the added noise and the simulated UHF signals are opposite in phase, and the wave-fronts of the noisy UHF signals are not extracted correctly.

**Figure 6:**
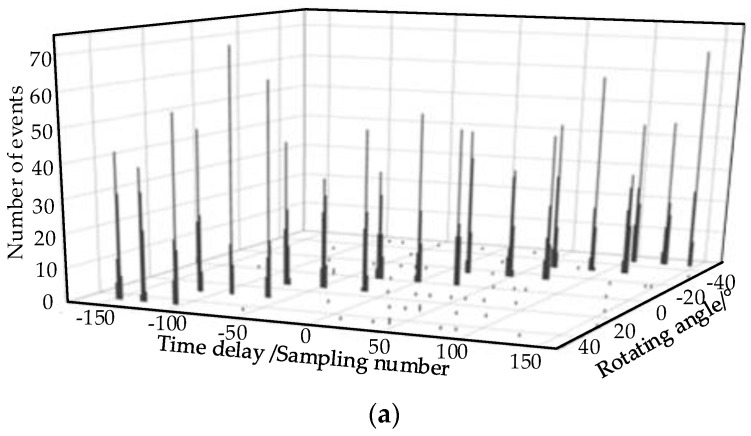

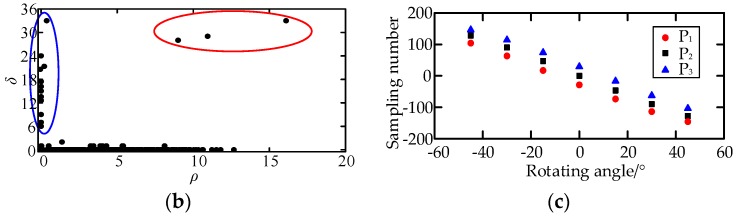
The TDs and decision diagram at O_2_ when SNR = 5 dB. (**a**) The TDs at the measurement point O_2_; (**b**) The decision diagram when *α_j_*= 0°; (**c**) The separated TD center sequences at O_2_.

The 300 TDs corresponding to three PDs with each rotating angle were obtained. Taking signals received at O_2_ as an example when SNR=5 dB, the TDs are shown in Figure 6a. The decision diagram of the 300 TDs at 0° is listed in Figure 6b. The density and distance of the three samples enclosed by the ellipse with red line are both the maximum; therefore, three TD centers are obtained. Several samples enclosed by the ellipse with blue line have the singular values, and it indicates that the distances of samples are the maximum, but the densities are small. The TD centers are 0, −29 and 28 in sampling number. Therefore, all the TD centers are all obtained and the singular TDs are excluded by the decision diagram correctly. With the separation method in Section 2.3, the parameters *γ* and *θ* are obtained at O_2_, as shown in Table 2, and the TD center separation results of O_2_ are shown in Figure 6c. With the separated TD center sequences, the direction angles of the three PDs at each measurement points are calculated by Equations (2) and (3). Separating the direction angles by the method proposed in Section 3, the results are shown in Table 3. By the error probability localization method, the locations of the three simulated PD source are obtained and listed in Table 4. Meanwhile, the separation rate is calculated, which is the percentage of signals that are correctly separated. The separation and localization results calculated by the previously reported method are listed in Table 5. It indicates that the separation rate is improved from 71% to 95% by the proposed method when SNR = 5 dB. The three PD sources are located in 3-D by the proposed method, and the maximum error is 0.16 m, while the localization result in [7] are the coordinates on the 2-D plane, and the errors on 2-D are still larger than the errors in 3-D of the proposed method. Compared with the localization results in [7], the separation rate and localization accuracy are all improved effectively.

From Table 4, the trend of the error is that, with the decrement of SNR, the error increases. This is because the errors of the TD increased as a result of the value increment of the noise. However, the localization of P_2_ is an exception when SNR = 5 dB. The reason could be observed in Table 3. The *σ_φ_* is larger than *σ_θ_*, therefore, the localization error mainly exists on the *z*-axis. The errors of P_1_ and P_3_ on *φ* are all negatives, which amplifies the error. However, the errors of P_2_ on *φ* are −1.6°, 0.7°, and 0.7°, and the errors of the positive and negative value counteract with each other. Therefore, the localization accuracy is improved.

### 4.3. The Simulation Result of Case 2

In this case, the parameters of P_2_ and P_3_ are the same, which is different from reference [7]. With the same localization method, four UHF sensors were used to locate the three PD sources. The coordinates of the four UHF sensors were #1 (0.5, 0.5, 1), #2 (3.5, 0.5, 1), #3 (3.5, 4.5, 1) and #4 (0.5, 4.5, 1) in meters. The UHFsignals received by #3 sensor were used as the samples to separate the three PD source, and white noise and communication frequency interference were added as the Section 4.2. The separation rate calculated by the proposed method and the previously reported method with different SNRs are shown in Table 6. It shows that the previous reported method had a much poorer separation rate. In the result the PD sources P_2_ and P_3_ wereclassified as one class. The reason was that the differences between frequency spectrums P_2_ and P_3_ were too small, and the five characteristic parameters were corrupted.

## 5. Field Test in Substation

### 5.1. Test Arrangement

**Figure 7:**
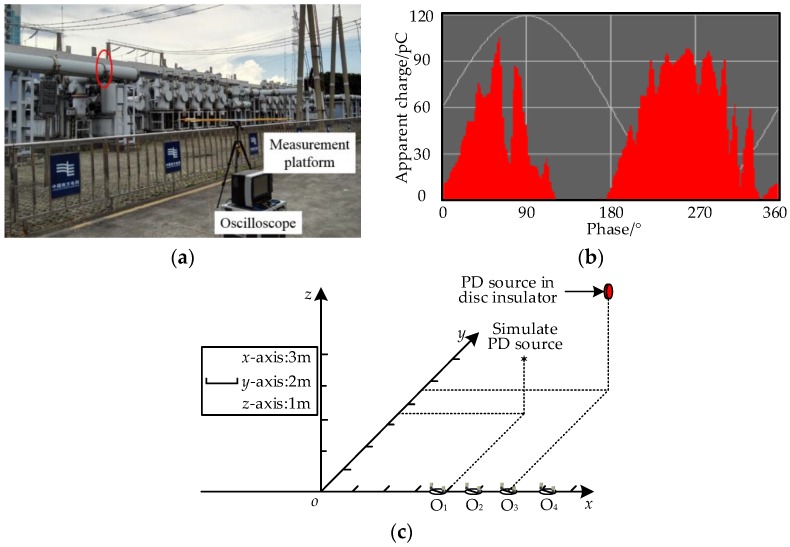
The field test site arrangement and the on-line monitoring result of the disc insulator PD. (**a**) The field test site and the test arrangement; (**b**) The on-line monitoring result; (**c**) The coordinate system of the field test site arrangement.

To verify the effectiveness of proposed method, a field test in a 220kV substation was carried out. The field photograph is shown in Figure 7a. The Vivaldi antenna [19] was adopted as the directional antenna. A LeCroy WR740Zi oscilloscope was used as the signal-acquisition system with the band width of 4 GHz and a maximum sampling rate of 40 GS/s. The antennas were connected to the oscilloscope through the identical coaxial cables. There were two PD sources in the substation. The first was a PD source located in a disc insulator with the air-gap defect, the location is enclosed by the ellipse with red line in Figure 7a, and the result of on-line monitoring system is shown in Figure 7b. The second was a simulated PD source. It consisted of point-point electrodes with air gap (the gap length is 5 mm) and a Tesla coil to simulate the PD caused by the poor electrical contact. The simulated PD source was controlled manually to simulate the intermittent discharge. In the substation, the coordinate system was established. The location range of the disc insulator was {*x*|*x*∈(17.3, 17.4)}, {*y*|*y*∈(8.55, 9)}, {*z*|*z*∈(2.6, 3)} in meters. The coordinate of the second PD source was (12, 5, 1.5). Four measurement points were selected, O_1_ (10, 0, 0), O_2_ (14, 0, 0), O_3_ (18, 0, 0) and O_4_ (22, 0, 0); the locations of the PD sources and measurement points are shown in Figure 7c. At each rotating angle, 150 UHF signals were recorded by the oscilloscope with the width trigger type.

### 5.2. The Separation and Localization Results

**Figure 8:**
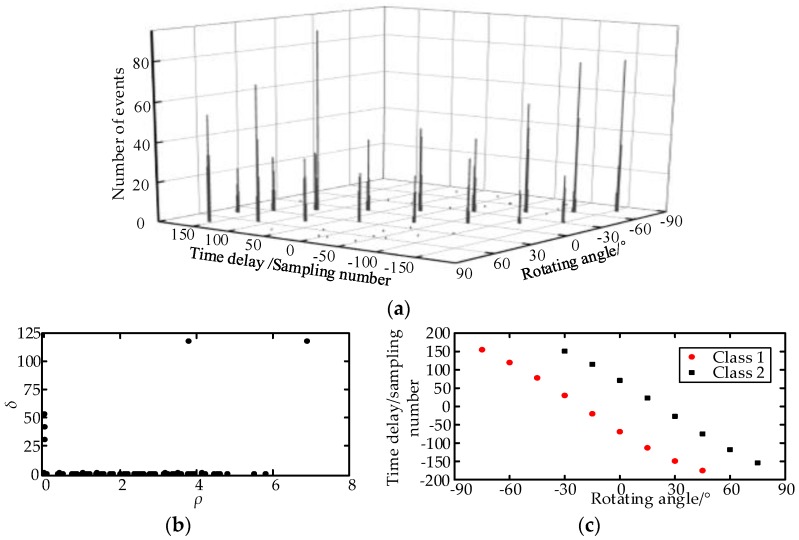
The TDs and decision diagram at O_2_. (**a**) The TDs at the measurement point O_2_; (**b**) The decision diagram when *α_j_*= −30°; (**c**) The separated TD sequences at O_2_.

**Figure 9:**
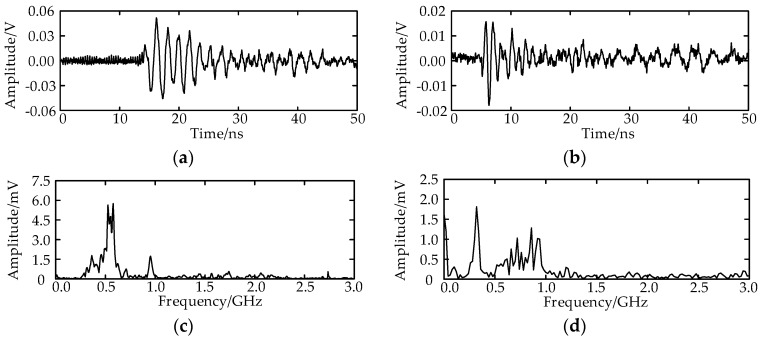
The UHF signals received at O_2_; (**a**) The waveform of the UHF signal radiated from the first PD source; (**b**) The waveform of the UHF signal radiated from the second PD source; (**c**) The spectrum of the UHF signal radiated from the first PD source; (**d**) The spectrum of the UHF signal radiated from the second PD source.

The TDs are calculated as shown in Figure 8a. Figure 8b illustrates the TD sample decision diagram of the measurement point O_2_ at −30°. The separation result of the TD center sequence is listed in Figure 8c. There are several singular TDs, the reason for which is that the amplitude of the field noise increased suddenly, which triggered the oscilloscope, and the field noise was recorded. Total 147 effective UHF signals are obtained, and they are separated into two classes. The first class has93 UHF signals, and the TD center is 31 sampling numbers. The second class has 54 UHF signals, and the TD center is 151 sampling numbers. The real discharge number of the two PD sources is unknown; therefore, the separation rate cannot be obtained. The UHF signals and the spectrums of the two PD sources are shown in Figure 9.

**Figure 10:**
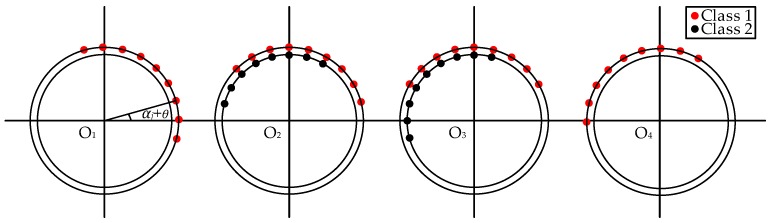
The TD distributions at each measurement point.

The TDs at each measurement point and each rotating angle are calculated and separated; the separation results are shown in Figure 10. At O_1_ and O_4_, only the first PD source is detected. At O_2_ and O_3_, the two PD sources are both detected. The directions of the two PDs are calculated by Equations (2) and (3), and the locations are obtained by the error probability localization method. The localization coordinate of the first PD source is (17.43, 8.54, 2.9) in meters. With the coordinate, the disc insulator can be found effectively. The localization coordinate of the simulated PD source is (11.92, 5.1, 1.64) in meters, and the error is 0.18 m.

## 6. Discussions

During PD measurements in a substation, the UHF signal is often interfered with by the field noise. In [7], the values of five frequency points were used as characteristic parameters to separate the multi-PD sources. To locate the PDs, the noisy UHF signal should be analyzed to extract the five frequency points. The five characteristic parameters may be corrupted by field noise, which reduces the separation rate. More seriously, if the frequency spectrums of multi-PD sources are the same, the separation rate drops sharply. In this paper, the TD distribution feature is used to separate the multi-PD sources, which avoids analyzing the waveform of the noisy UHF signal. Many TD estimation methods can suppress the field noise, and the TD estimation method used in this paper [11] is just one of them. Therefore, the separation rates are improved effectively.

The localization results in [7] were the 2-D coordinates. From [10,11], with the same sampling rate, the error of the distance between the measurement point and PD source is too large to locate the PD source, but the direction angles in 3-D are accurate. Therefore, by selecting several measurement points in the substation, the direction angles are obtained at different measurement points. With the multi-directions, the problem that the error on *z*-axis is too larger can be solved.

In the field test, the error of the second PD source is greater than the first. The reason is that the UHF signals were detected at only two measurement points. If more directions are obtained, the accuracy can be improved effectively.

## 7. Conclusions

In this paper, a novel method for separating and locating the multi-PD sources is proposed. A simulated model with three PD sources was established and a field test was carried out to verify the method. The conclusions are summarized as follows:

(1) The separation rate is increased effectively. With the TD center extracted by the density peaks clustering method, the error TD is removed and the dispersion problem of TDs is solved. The TD sequence distribution feature is used as the characteristic parameter to separate the multiple PD sources. Therefore, the analysis of the noisy UHF signal’s waveform is avoided, which is the key of improving the separation rate.
(2) The localization accuracy is improved. Through rotating measurement at a point, the deficiency of the unidirection of the directional antenna is overcome, and an accurate direction between the PD source and the measurement point is obtained. With the multi-point measurement and error probability localization method, the PD source can be located in 3D.
(3) To verify the proposed method and compare with the previously published method, a simulation model with three PD sources was established by XFdtd. With the case of three different PD sources, the separation rate was increased, using the proposed method, from 71% to 95% when SNR = 5 dB, and the accuracy of the localization result was improved from 2-D to 3-D. With the case of the two same PD sources, the separation rate of the previously published method declined seriously, while the proposed method is not affected.
(4) A field test was carried in a 220 kV substation. An air-gap discharge in a disc insulator and a simulated PD source were used as the testing targets. The two PD sources were separated effectively with the TD distribution features and were located in 3-D accurately by the error probability of the directions. This indicates that the proposed method is effective in the field test.

## Figures and Tables

**Table 1:** The parameters of the three simulated PD sources.

Case	P_1_		P_2_		P_3_	
	Amplitude/A	Width/ns	Amplitude/A	Width/ns	Amplitude/A	Width/ns
Case 1	1	4	1.5	2	2	6
Case 2	1	4	2	2	2	2

**Table 2:** The characteristic parameters of the three measurement points when SNR = 5 dB.

Measuring Points	P_1_		P_2_		P_3_	
	*γ*	*θ*/°	*γ*	*θ*/°	*γ*	*θ*/°
O_1_	−181.3	−63.5	−180	−72.8	−176.4	−80.2
O_2_	−178.1	−80.8	−179.7	−90.2	−178.1	−99.2
O_3_	−182.2	−99.6	−180	−107.2	−186.7	−115.8

**Table 3:** The direction angles of the three measurement points when SNR = 5 dB.

Measuring Points		O_1_	O_2_	O_3_
P_1_(*θ*/°, *φ*/°)	Theoretical value	(63.4, 24.1)	(80.5, 26.3)	(−80.5, 26.3)
	Calculated value	(63.7, 22.5)	(80.7, 27)	(−80.5, 27.1)
	Error	(0.3, −1.6)	(0.2, 0.7)	(0, 0.7)
P_2_(*θ*/°,*φ*/°)	Theoretical value	(72.9, 23.8)	(−90, 24.8)	(−72.9, 23.8)
	Calculated value	(72.8, 24.4)	(−89.8, 26)	(−72.7, 25.7)
	Error	(−0.1, 0.6)	(−0.2, 1.2)	(−0.2, 1.9)
P_3_(*θ*/°,*φ*/°)	Theoretical value	(80.5, 26.3)	(−80.5, 26.3)	(−63.4, 24.1)
	Calculated value	(80.1, 25.9)	(−80, 25.4)	(−63.9, 23.1)
	Error	(−0.4, −0.4)	(0.5, −0.7)	(−0.5, −1)

**Table 4:** The separation and localization results by the proposed method with different SNRs.

SNR/dB	Separation Rate/%	P_1_Localization Result/m		P_2_Localization Result/m		P_3_Localization Result/m	
		Coordinate	Error (3-D)	Coordinate	Error (3-D)	Coordinate	Error (3-D)
25	100	(1.99, 6.47, 3.05)	0.06	(2.97, 6.02, 2.94)	0.07	(1.01, 5.99, 2.97)	0.03
10	98.7	(2.02, 6.46, 3.07)	0.08	(3.03, 5.99, 3.1)	0.1	(0.96, 6.04, 3.06)	0.08
5	95	(1.99, 6.44, 3.15)	0.16	(2.99, 6.04, 3.02)	0.04	(1.02, 6, 2.9)	0.1

**Table 5:** The separation and localization results using previously reported method at different SNRs.

SNR/dB	Separation Rate/%	P_1_Localization Result/m		P_2_Localization Result/m		P_3_Localization Result/m	
		Coordinate	Error(2-D)	Coordinate	Error(2-D)	Coordinate	Error(2-D)
25	84	(2.13, 6.67)	0.21	(2.85,6.12)	0.19	(1.07,6.09)	0.11
10	77	(2.19, 6.73)	0.30	(2.79, 6.24)	0.32	(0.87, 6.16)	0.21
5	71	(2.27, 6.84)	0.43	(2.67, 6.29)	0.44	(1.25, 6.19)	0.31

**Table 6:** The separation results by the proposed method and the published method.

SNR/dB	Separation Rate Calculated by the Proposed Method/%	Separation Rate Calculated by the Previously Reported Method/%
25	100	68
10	98.3	62
5	95.3	56

## Abbreviations

The following abbreviations are used in this manuscript:

PD	Partial discharge
UHF	Ultra-high frequency
SNR	Signal noise ratio
TD	Time delay
TDOA	Time difference of arrival
